# Enhancing Effects of Environmental Enrichment on the Functions of Natural Killer Cells in Mice

**DOI:** 10.3389/fimmu.2021.695859

**Published:** 2021-07-28

**Authors:** Run Xiao, Seemaab Ali, Michael A. Caligiuri, Lei Cao

**Affiliations:** ^1^Department of Cancer Biology and Genetics, College of Medicine, The Ohio State University, Columbus, OH, United States; ^2^The Ohio State University Comprehensive Cancer Center, The James Cancer Hospital and Solove Research Institute, Columbus, OH, United States; ^3^Medical Scientist Training Program, The Ohio State University, Columbus, OH, United States; ^4^Department of Hematology & Hematopoietic Cell Transplantation, City of Hope National Medical Center and the Beckman Research Institute, Los Angeles, CA, United States

**Keywords:** environmental enrichment (EE), immune function, natural killer (Nk) cell, BDNF, HPA axis, sympathetic nervous system, cancer

## Abstract

The environment of an organism can convey a powerful influence over its biology. Environmental enrichment (EE), as a eustress model, has been used extensively in neuroscience to study neurogenesis and brain plasticity. EE has also been used as an intervention for the treatment and prevention of neurological and psychiatric disorders with limited clinical application. By contrast, the effects of EE on the immune system are relatively less investigated. Recently, accumulating evidence has demonstrated that EE can robustly impact immune function. In this review, we summarize the major components of EE, the impact of EE on natural killer (NK) cells, EE’s immunoprotective roles in cancer, and the underlying mechanisms of EE-induced NK cell regulation. Moreover, we discuss opportunities for translational application based on insights from animal research of EE-induced NK cell regulation.

## Introduction

The environment of an organism can convey a powerful influence over its biology and physiology. How different environments influence health outcomes is studied in part within the field of stress research. For most applications, stressors can be categorized as one of two types: distress and eustress. “Stress” most commonly refers to distress, a negative or maladaptive state which poses a risk to health and well-being (1). Relatively speaking, eustress is a less commonly used term that relates to milder and/or briefer challenges, which are manageable and induce a positive or healthy, adaptive response (2, 3). Most stress research has focused on the impact of distress on health. The impact of eustress, which is associated with health benefits, has been attracting attention in recent years.

One of the most widely used eustress models in laboratory rodents is environmental enrichment (EE). The beneficial effects of EE have been explored through a long history of research. Initially, EE was used extensively in neuroscience as a model for neurogenesis, brain plasticity, and learning and memory, as well as a tool for the treatment and prevention of neurological and psychiatric disorders (4–6). Apart from the studies in earlier years identifying the effects of EE on the nervous system, follow-up research has shown that EE also exerts a significant influence on other systems such as the endocrine system and the immune system (7–9). Accumulating evidence has shown that EE profoundly influences the immune system. Here we summarize the components of EE and EE’s effects on immune cells. We focus on the impact of EE on natural killer (NK) cells, in particular the EE-induced protective roles of NK cells in cancer. We also discuss the underlying mechanisms of EE-induced NK regulation, and briefly consider the potential implications of EE research for translational interventions in human health and disease.

## What is Environmental Enrichment?

Donald Hebb was the first to propose EE as an experimental tool as early as the late 1940s (10–12). Hebb performed foundational studies to discover the connection between EE and improvement in cognition and behavior. Although in these initial studies the beneficial effects of EE were examined from the perspective of the nervous system, follow-up studies have found that EE’s effects are more systemic and complicated than initially conceived.

**Figure 1:**
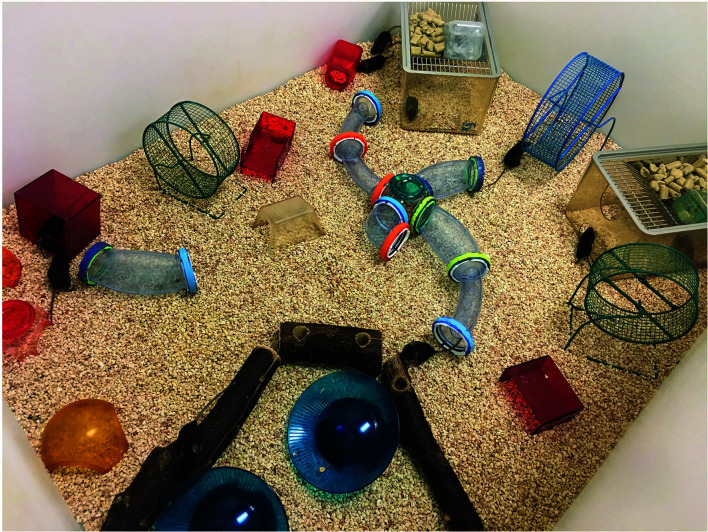
A typical EE setting. The precise paradigm implemented in studies of EE varies both from lab to lab, but key features are generally similar. In contrast to standard housing, EE is a bigger cage supplemented with running wheels, tunnels, igloos, huts, retreats, wood toys, a maze, and nesting material. The supplements in EE cage are exchanged or rearranged once a week over the period of the experiments.

In the experimental laboratory setting, an “enriched environment” is compared with a “standard environment (SE)”. Although the settings of EE vary in different laboratories, generally speaking, they have the same components. Compared with SE, EE is defined as housing enrichments with increased space, physical activity, and/or social interactions that confer enhanced sensory, cognitive, motor, and social stimulation (4). In our studies (**Figure 1**), we house control mice in standard laboratory cages of 30.5 cm × 17 cm, at 5 mice per cage with nesting material. In comparison, EE mice are housed in a larger cohort of 10-20 mice in a bigger cage, 1.5 m × 1.5 m, supplemented with running wheels, tunnels, igloos, huts, retreats, wood toys, a maze, and nesting material (13). In addition, the supplemental items in EE cages are exchanged or rearranged once a week over the period of the experiments to provide for increased novelty and complexity in the space.

## The Major Components of Environmental Enrichment

EE consists of environments which provide physical enrichment (the cages contain running wheels for voluntary exercise), social enrichment (the animals are placed in larger groups per cage), and cognitive and sensory enrichment (the animals are provided with a variety of objects to interact with, tunnels, and spaces to navigate) (14). These three components are key features of an EE for rodents. Therefore, the question is often raised as to which components are important for eliciting specific beneficial effects of EE. The importance of individual components of EE has been tested previously. Our studies and studies from other groups support the view that a single contributing factor of EE cannot be easily isolated for most of EE’s beneficial effects (15–18). Instead, the components of EE work together to exercise overlapping, unified, or emergent effects on rodents living within it. Any one of these components cannot account for all of the effects of EE, thereby supporting the description of EE as “a combination of complex inanimate and social stimulation” (19). The individual impacts of physical enrichment, social enrichment, and cognitive and sensory enrichment, as they compare to the impact of combined EE, are described below.

### Physical Enrichment

Similar to EE, physical exercise has been shown to enhance immune function (20) and to decrease body fat (21). To investigate whether physical exercise could alone account for the EE-induced phenotypes, we provided mice with access to voluntary wheel running. Wheel running led to physiological changes including reduced adiposity, and increased lean mass similar to that observed in the EE mice (15, 16). However, EE and wheel running also exhibited several differences as described below. EE was associated with altered adipokine levels in serum characterized as a decrease of leptin while an increase of adiponectin. The sharp drop of leptin was identified as a key mediator of EE’s anticancer effect (15). Runners displayed a pattern of adipokines quite distinct to that of EE with no change in serum leptin level and a decreased serum adiponectin. Moreover, running influenced gene expression in the arcuate of hypothalamus with a pattern that was qualitatively different than EE. In contrast to the EE mice whose brain-derived neurotrophic factor (*Bdnf)* expression was increased 3-fold at 4 weeks of EE, running did not upregulate *Bdnf* significantly. Instead, the two orexigenic peptides neuropeptide Y (*Npy*) and agouti-related peptide (*Agrp*) were increased by wheel running. Lastly, although enhanced innate immune cytotoxicity was observed in both runners and EE mice, exercise alone did not significantly reduce tumor weight as EE achieved. These differences were not due to higher physical activity in EE. In fact, mice living in cages with free access to running wheels ran approximately 2 km per day. In comparison, EE mice traveled a mean total distance of 0.64 km per day, which is approximately one third of distance the runners traveled (15). Additional studies (16, 18) further support the notion that physical exercise alone is insufficient to account for EE-induced phenotypes, including tumor resistance, although it likely contributes to these outcomes.

### Social Enrichment

Social enrichment is a significant contender to account for many of the effects of EE. In fact, some studies identify social enrichment as capable of reproducing specific neurobiologic effects of EE. For example, Moreno-Jiménez and colleagues have shown the social component of EE to be as potent a stimulus of adult hippocampal neurogenesis as EE (22). However, a study by Rosenzwig et al. lead to the opposite conclusion (19). In that study, authors tested whether mere group living (social stimulation) is the major contributor of EE to measures of brain anatomy and brain chemistry that develop in EE animals. In detail, one control group of 12 male rats were assigned to a large cage without stimulus objects (19). These stimuli were the only difference when compared with the EE setting cohort. After one-month duration, measures were taken of weights of brain regions, RNA and DNA contents of regions of cerebral cortex, and acetylcholinesterase activities of brain regions. Although the cage size and the number of rats housed together were the same between the EE group and corresponding control group, the results of the cerebral measures differ considerably as a consequence of the inanimate stimulus (19). These observations suggest living in the same-sized group and in the same-sized cage is less effective at altering the brain than doing so with inanimate enrichment. Taken together, social enrichment alone is not sufficient to reproduce all of the cerebral effects of EE (11, 19).

### Cognitive and Sensory Enrichment

There are few studies to directly compare the specific effect of cognitive and sensory enrichment with the typical complex EE setting. We can distinguish the differences between them from separate parallel studies which have tested the impact of either cognitive stimulation or EE on progression of the same disease in murine model. Specifically, Jankowsky et al. showed that EE conferred a beneficial effect in a mouse model of Alzheimer’s disease by mitigating cognitive deficits (23). In sharp contrast, environmental novelty alone exacerbates stress hormones and Aβ pathology in an Alzheimer’s model (24). In addition, it has been shown that observing an enriched environment without being allowed active participation, does not elicit the cerebral effects of EE (25). These contrasting results suggest cognitive enrichment alone cannot account for EE. Altogether, these studies have revealed that no single variable can account for the multifaceted consequences of enrichment. Instead, each of these components of EE interact in complex ways.

Other factors which we have not listed also contribute to the effects of EE. Of note, the length of an EE exposure can greatly impact observed outcomes: the phenotypes of EE do not show up at the same time. The durations for EE exposure used in mouse studies are mostly from one week to a couple of months. EE is also delivered either continuously, as in continuous EE housing, or at interval exposures, such as daily for a few hours. EE increases adipose NK cell percentage after two-weeks exposure (26). In contrast, EE decreases systemic leptin levels after a longer duration, more than four weeks (15). In addition, 5-week EE exhibits stronger anticancer effect than 3-week EE. These data suggest duration of EE exposure impact the EE’ phenotype.

## Environmental Enrichment Influences NK Cell Function

Increasing evidence has shown that EE produces notable effects on the phenotype and function of immune cells in mice. Take T cells for example: exposure to an EE for merely 1 week is sufficient to increase the frequency of single positive (SP) CD8^+^ T cells and decrease the frequency of SP CD4^+^ T cells, thereby decreasing the CD4:CD8 ratio in both thymus and spleen (27, 28). In addition to influencing adaptive immune cells, EE also impacts innate immune cells. Analysis of peripheral blood samples from mice after a two week EE exposure shows no difference in total numbers of blood leukocytes or individual leukocyte populations (29). However, EE mice display a significant difference in their relative proportions of leukocyte populations, characterized by a higher proportion of neutrophils and a correspondingly lower proportion of lymphocytes compared to SE mice, suggesting myeloid cells are responsive to EE (29). Studies of EE’s effects on macrophages have been reported, as well (30). Here we focus on the effects of EE on NK cell function.

### Impact of EE on NK-Cell Maturation

NK cells are innate immune cells that are sensitively responsive to internal or external stimuli (31, 32). Mouse NK cells undergo a maturation process that is defined by expression of the surface markers CD27 and CD11b. Developing NK cells are classified as precursor (stage I, CD11b^low^ CD27^low^), immature (stage II, CD11b^low^ CD27^high^), proinflammatory (stage III, CD11b^high^ CD27^high^), or cytotoxic (stage IV, CD11b^high^ CD27^low^) (33). As NK cells progress through these stages of maturation, they gradually lose their proliferative capability (34). EE promotes the terminal maturation of NK cells both centrally in the bone marrow and peripherally in the spleen and blood of tumor-free mice (35). NK cells with enhanced maturation have also been found in tumor-bearing mice after EE exposure (35). The maturation of NK cells is crucial for the acquisition of optimal functional activity and for their immune response to tumors. As defined before, NK cells in stage II (CD11b^low^ CD27^high^) and stage III (CD11b^high^ CD27^high^) show the best capability of cytokine secretion, whereas NK cells in stage IV (CD11b^high^CD27^low^) exhibit the greatest cytolytic function (36).

### Impact of EE on NK-Cell Proliferation

EE increases CD27^+^ immature and proinflammatory NK-cell proliferation in the bone marrow and blood of tumor-free mice (35). In contrast, EE increases splenic CD27^+^ immature and proinflammatory NK-cell proliferation in tumor-bearing mice, which in turn have a lower tumor burden. In agreement, splenectomy abrogates the EE-induced tumor-resistant phenotype (35) implicating splenocytes in tumor resistance. EE also impacts the recovery of NK cells after depletion (37). We observed a faster NK-cell recovery in the spleen, bone marrow and blood following depletion using anti-asialo-GM1 antibody (37).

### Impact of EE on NK-Cell Cytotoxicity

Consistent with enhanced NK-cell maturation and proliferation, EE enhances NK-cell cytotoxicity in both tumor-free mice and tumor-bearing mice (15, 38). Song et al. report that NK-cell-mediated immunity plays a critical role in EE–induced tumor inhibition in murine tumor models of pancreatic cancer and lung cancer (39). They identified certain markers associated with higher cytotoxic activity of tumor-infiltrating NK cells from EE mice. The expression of NKG2D, an activating cell surface receptor (40), on tumor-infiltrating NK cells is higher in EE mice (39). In addition, the expression of CD107a, a functional marker of NK-cell activation and cytotoxicity (41, 42), on tumor-infiltrating NK cells from EE mice is also significantly increased (39). Interestingly, a recent report shows that minimal cage enrichment provided by placing one mouse igloo within a standard cage can enhance the activation of NK cells relating to antitumor immunity in a murine ovarian cancer model (43). Collectively, these studies suggest a positive modulating effect of EE in promoting NK-cell maturation and cytotoxicity.

### Impact of EE on NK-Cell Infiltration Into Tumors

As discussed above, EE exposure not only affects immunity under normal conditions, but also produces robust immune regulation under pathologic conditions. Song et al. reported that EE promoted tumoral infiltration of NK cells instead of T lymphocytes in a mouse model of pancreatic cancer (39). Both immunohistochemical analysis and flow cytometry analysis demonstrate more abundant tumoral NK cells from EE mice (39). EE upregulated the cell surface expression of the chemokine receptor CCR5 in blood- and tumor-derived NK cells, which plays an important role in the enhancement of tumoral NK-cell infiltration (39). In a murine model of glioma, EE increased NK cell accumulation at tumor site mediated by IL-15 (30).

### Impact of Experimental Context for EE on NK Cell Function

Contextual factors, such as age, sex, and obesity, will play a major role in outcomes for any EE clinical interventions (as with any clinical intervention). These factors are known to have significant effects on immune function and particularly on NK cells (44–47). In animal studies modeling subgroups where deficits or health hazards are more pronounced (disease models for autism spectrum disorder, post-stroke, obesity, age-related disease, etc.), EE appears to have more substantial benefits. In these contexts, EE tends to improve outcomes and bring animals closer to their model control baselines – although the disease model and the context being studied matters significantly. With respect to NK cell activity, one animal study has identified a protective effect of a minimal type of EE on NK cell function during aging (8). Very old mice 20-22 months old show reduced NK cell-mediated lysis of tumor cells compared to adult and older middle-aged mice. This NK cytotoxicity deficit can be prevented by exposure to EE for 2-4 months starting at 20-22 months old.

## Key Mediators for Environmental Enrichment’s NK-Cell Regulation

Much of the research into the immune system or into the nervous system considers these systems separately, exerting their own functions independently. However, accumulating evidence has demonstrated substantial cross-talk between the nervous system and immune system, capable of inducing reciprocal action on one another (48, 49). Specifically, neurons can produce immune cytokines that act on immune cells, and immune cells can produce neurotransmitters and neuropeptides that act on neurons. Endocrine system also communicates and provides feedback between both the nervous system and the immune system through adipokines, cytokines, hormones, and other signaling molecules. Therefore, it is not surprising that EE, initially utilized as a neurological model, can robustly impact the immune system. Next, we summarize the key mediators involved in EE-induced immune regulation including NK cells.

### BDNF

Our team was first to identify hypothalamic BDNF as a key regulatory molecule in EE (15, 18). At an early time point of 2 weeks in EE, *Bdnf* upregulation was detected in the arcuate and ventromedial/dorsomedial hypothalamic nuclei (15). Importantly, hypothalamic gene delivery of BDNF mediated by an adeno-associated virus (AAV) recapitulated the mild activation of the sympathetic nervous system (SNS) and the hypothalamic-pituitary-adrenal axis (HPA) that are critical features of EE’s phenotype. Consequently, this treatment reproduced the EE-induced anticancer effects seen in multiple murine cancer models. Furthermore, overexpression of hypothalamic BDNF reproduced EE-induced NK-cell phenotypes characterized with a high percentage of late-stage maturity of NK cells, whereas knockdown of hypothalamic BDNF abrogated EE-induced NK modulation (37). These data suggest hypothalamic BDNF is a mediator molecule for EE-induced NK cells. In addition, we reported hypothalamic BDNF was required for EE-induced T cell regulation in both primary and secondary lymphoid tissues (27, 28). Together these observations suggest hypothalamic BDNF as a critical immunomodulatory molecule in the brain, which increases in response to EE and which induces downstream immune changes. We have also investigated other molecules which are regulated in the hypothalamus of EE mice, none of which can recapitulate the peripheral phenotypic changes induced by EE in the way that hypothalamic BDNF can (50). This evidence strengthens the view of hypothalamic BDNF as a master gene coordinating the metabolic and immune regulatory effects of EE. Importantly, this pathway differs from the local mechanisms thoroughly elucidated by Garofalo et al., in which BDNF produced in the brain of EE mice modulates NK cell activation through IL-15 expressed by brain microglia in the context of glioma (51). It is likely that other molecules act selectively in extrahypothalamic brain regions, such as prefrontal cortex, contributing to EE’s immune modulatory effects, warrant of further investigation.

### SNS

A large body of stress research implicates two nervous system or neuroendocrine pathways which exert a regulatory effect on the immune system: the sympathetic nervous system (SNS) and the hypothalamic-pituitary-adrenal (HPA) axis (52, 53). EE can serve as a model of eustress—mild and manageable stress associated with benign or beneficial adaptations (54). EE functions through these two arms in order to regulate immune function. Through the SNS, all primary and secondary immune organs receive substantial innervation from sympathetic postganglionic neurons (55, 56). Norepinephrine (NE) binds to either α- or β-adrenergic receptors expressed on the surface of immune cells including NK cells, which induces changes in gene expression of various immune-cell-derived factors (55, 57). In agreement, we observed serum NE levels trending upward in EE mice (15). Studies using nonselective β blocker administration inhibiting the SNS have shown that intact SNS is required for EE-induced NK-cell and T-cell phenotypes in the spleen (27, 39). Specifically, 6OHDA-induced sympathectomy eliminates the enhanced cytolytic activity and the increased expression of NKG2D and CCR5 in NK cells from EE mice (39), suggesting β-adrenergic receptor signaling regulates gene expression in NK cells that is related to NK cell function.

### HPA Axis

Glucocorticoids play an important role in regulating the inflammatory and immune response. Glucocorticoids are used in medicine to treat diseases caused by various inflammatory and autoimmune disorders (58). All immune cells express glucocorticoid receptors (GR) and are responsive to glucocorticoids released by activation of the HPA axis, though GR expression and responsiveness vary (59). In our studies, the mRNA level of corticotropin-releasing hormone (*Crh*) from EE mice was elevated in the hypothalamus (18). Glucocorticoid levels in the serum of EE mice were also mildly elevated, approximately 50% (15, 28). Our data from thymocyte-specific GR knockout mice or adrenalectomized mice demonstrated that the EE-induced T-cell phenotypes in the thymus and spleen are dependent on the activation of the HPA axis (27, 28). Moreover, our preliminary data shows that adrenalectomy also abolishes the enhancement of NK cell maturation in EE, suggesting an important role of the HPA axis in EE-induced NK-cell phenotypes. These data support a notion that HPA axis can exert a positive regulation on NK cell functions although glucocorticoids are shown to have an inhibitory effect on immune cell functions. We agree with the idea of Capellino et al. that the effects of glucocorticoids on NK cells depends on the concentration of glucocorticoid and the specific biological context (60). Of note, this does not mean that EE-induced immune regulation in every organ throughout the body is dependent on the HPA axis. For example, our unpublished data has also shown that EE increases the frequency of NK cells in adipose tissue, which is not abrogated by adrenalectomy. These data suggest that EE fine-tunes innate and adaptive immunities through a coordinated regulation of both arms of stress responses in a tissue-specific manner.

### Adipokines

EE robustly reduces adiposity with little effect on body weight and enhances energy expenditure, leading to anti-obesity phenotype. The profound adipose remodeling induced by EE is also mediated by hypothalamic BDNF through a specific brain-fat axis, the hypothalamic-sympathoneural-adipocyte (HSA) axis. One consequence of the HSA axis activation is the alteration of adipokine levels in the circulation (15, 18, 54). EE regulates the expression and release of two major adipokines leading to a large decrease in serum leptin level up to 75% (15), whereas an increase in adiponectin by ~30% after 4 weeks of EE (15). Numerous studies have investigated the connection between these adipokines and regulation of immune cells (61–63). Evidence has shown decreased leptin and increased adiponectin are correlated with an anti-inflammatory state. Of note, EE has been associated with suppression of pro-inflammatory marker expression (16, 64, 65). However, the question of how these adipokines impact immune cells, including NK cells, in the context of EE remains unexplored and needs further investigation.

### Cytokines

EE exposure reduces tumor size and the proliferation rate of glioma cells, and improves survival in an orthotopic glioma implantation model (30). Mechanistically, one of the pathways responsible for this benefit is EE-induced NK cell accumulation and activation mediated by an increase of IL-15 (30). IL-15 plays an important role in NK cell development and maturation (66, 67). Given that NK cells exhibited an enhanced maturation phenotype in EE mice, we speculate that EE-induced IL-15 may promote the maturation of NK cells.

Cytokines are involved in physical exercise-induced NK cell activation and inhibition of tumor growth. However, the underlying mechanism may be distinct between EE and physical exercise alone. For example, exercise-induced muscle-derived IL-6 is involved in NK cell mobilization and redistribution during voluntary running (68). In contrast, in our preliminary data, we have not observed higher plasma IL-6 or higher expression of *Il6* in muscle from EE mice. Cytokine profile analysis from serum samples is worth further investigation in order to identify further altered cytokine profiles between SE and EE mice that may mediate other effects on immune regulation. How cytokine alterations after EE affect immune cells is less well understood and should be explored.

### Eustress and Distress

From the key mediators of EE, we note that some of them, such as the SNS and the HPA axis, are also involved in distress. Differences between EE as eustress and conditions of distress help us understand their outcomes. There are two aspects to consider. On one hand, certain key mediators are unique to EE. For example, higher expression of hypothalamic BDNF and decreased level of leptin are associated with EE, but not distress (15). On the other hand, the degrees of change in shared mediators stress responses are also different. Only a trend of increase in serum NE and a moderate increase (~50%) of glucocorticoids are observed in EE mice (15, 28). In contrast, NE level and glucocorticoids increase by more than 2-fold in distress models (69, 70). Therefore, we expect the extent of change in NE and glucocorticoid level is regulated by the valence of a stress response.

The differences in these underlying mechanisms of eustress and distress account for their divergent effects on NK cells. As we lay out above, EE generally exerts a positive regulatory action on NK cell function, by promoting the proliferation and terminal maturation of NK cells and enhancing NK cell cytotoxicity. In contrast, a prolonged stressor such as restraint stress diminishes NK cell function and suppresses expression of the cytokine genes involved in the recruitment and activation of NK cells (71). Previous human NK cell studies have primarily identified chronic, prolonged distress will diminish NK cell function, and brief, acute distress may activate and mobilize NK cells – likely through pathways overlapping with EE (72).

## Implications of Environmental Enrichment for Human Health

**Figure 2:**
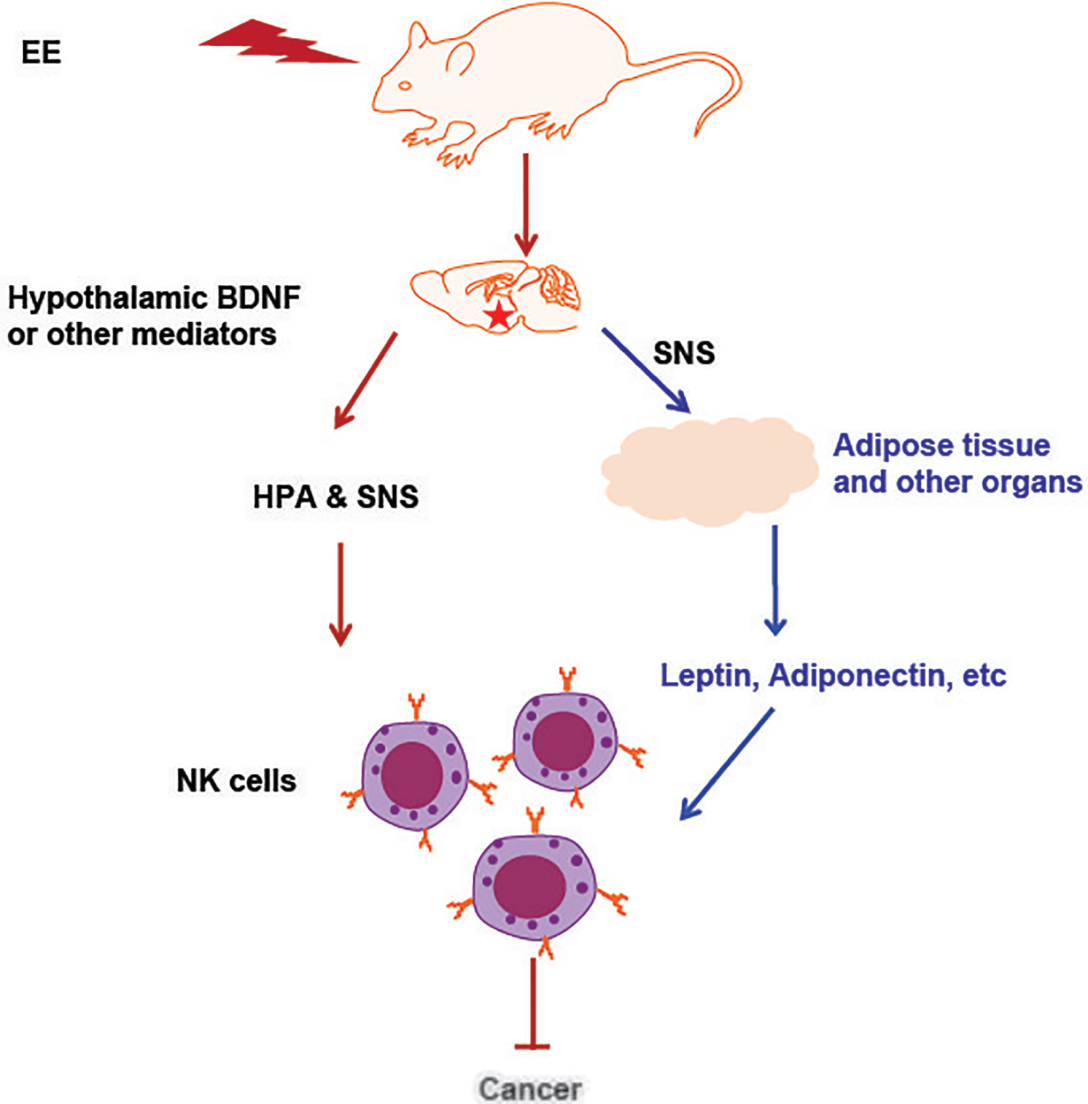
An illustration of NK cell activation and cancer inhibition induced by EE. The induction of hypothalamic BDNF or other brain mediator molecules in response to environmental stimuli leads to the activation of the SNS and HPA axes, which can directly act on NK cells. Another pathway is that EE impacted organs with endocrine function secrete adipokines, cytokines or hormones and subsequently act on NK cells that residing in the organ. The adipokines or cytokines also enter circulation, reach the distant organs and regulate NK cells there. All these effects contribute to EE induced immune protection against cancer and other diseases.

As we have explored, EE is a housing model that confers beneficial and protective effects on animal health (illustrated in **Figure 2**). How then can this understanding of EE be applied to human health? Concerns frequently arise related to the translatability of EE research to clinical settings, which we will briefly address here. This topic is discussed elsewhere, as well as in these reviews (73–75).

At the forefront among them is the concern about what “enrichment” entails in the context of humans and human disease. As described above, EE is species-specific and multifactorial. Many beneficial effects likely result from the overlap of several components. Interventions designed to elicit species-typical behavior for humans through enrichment may need to address: culture, art/music, higher order social structures, kinship/relationships, life routine, living space, and diet, just to name a few. These are each worth addressing and clearly have complex impacts on health outcomes. It is not clear that these dimensions of human experience correspond exactly to the features of EE known to act in mouse models, but they do relate to features such as sensory and spatial navigation, cognitive demand, social interaction, and physical activity. Engaging these is generally believed to be beneficial for our health as non-pharmaceutical interventions. Our work and others’ have shown that there exist robust biological pathways, otherwise not at play in standard environments, which are activated as a result of EE.

Another concern that arises is whether variation in animal exposures to components of EE during EE diminishes its validity as a model. Differences in outcomes may arise based on different experimental EE paradigms or different lived experiences among animals within any complex social and physical environment. This concern has been identified repeatedly, and studies addressing it frequently show reliability and reproducibility in EE (76–78). Basic standard environment features like bedding, housing density, temperature, and ambient noise all contribute to outcome variation with much less concern from the scientific audience, perhaps due to less awareness about welfare and husbandry needs (79). Humans are also varied, in terms of their genetics, their experiences, and their life history. Some researchers have proposed that because of the complexity and variation of human experiences, EE serves as a better baseline for our animal models when making inferences to human biology (6, 80).

The potential space for such EE to overlap in human laboratory studies and human clinical interventions is large. For example, given our knowledge of the impacts of environment and biology, changes to the environment of patients receiving care could potentially be implemented to improve well-being and health outcomes. Specific environments which may contribute enrichment for humans are still being explored. One study investigated features that constitute an “enriched environment” for people and identified associations between geographical properties and brain structure (81). In that study, living close to forests may be viewed as EE for humans. Due to the increased complexity of physical, social, and psychological factors contributing to the health of humans compared to mice, and given the wider scope of a humans’ life compared to that of a laboratory mouse, we expect that a kind of EE for humans includes but is not limited to geographical features. Despite advances in the understanding of EE, more studies need to be performed to apply an EE housing framework to human health.

Attempts to translate EE-mediated therapy that originated in animal models into humans remain in their infancy. Three clinical trials by Woo and Leon are among the first examples of direct translation of EE in animal models of autism spectrum disorder (ASD) to clinical studies (82–84). These randomized clinical trials have shown that EE in the form of Sensory Enrichment Therapy is capable of ameliorating symptoms of ASD. EE has also been shown to improve the symptoms of children with Rett syndrome (85). These studies provide a clear example of how preclinical studies in animal models of brain disorders can directly inform clinical investigations and trials.

Moreover, the health-promoting mechanisms of EE outlined above, including activating hypothalamic BDNF, or utilizing IL-15-induced NK cell maturation, can be translated into therapeutics. Hypothalamic BDNF plays a critical role in maintaining energy balance (86, 87). Genome-wide association studies have found that BDNF is one of 18 genetic loci associated with body mass index (88, 89). Haploinsufficiency for BDNF or its receptor TrkB is linked with hyperphagia and obesity (90–92). We have developed a molecular therapy and demonstrated the efficacy and safety of hypothalamic BDNF gene therapy in diet-induced and genetic models of obesity as well as aging mice of normal weight (93–96). In light of hypothalamic BDNF mediating the immunomodulating effect of EE, it will be interesting to explore targeting hypothalamic BDNF as an immune therapeutic. With regard to IL-15, we have developed a novel rAAV vector to overexpress IL-15/IL-15Rα specifically in the adipose tissue. A single intraperitoneal injection of IL-15/IL-15Rα vector at low dose results in expansion of NK cell in adipose tissue and enhanced NK activity in tumor-bearing animals leading to survival benefit (97). In addition, EE enhances NK cell antitumor immunity *via* regulation of NKG2D and CCR5 (39), which may be utilized in the genetic engineering of NK cells for enhanced antitumor function. As more mechanisms underlying EE-induced NK regulation are found, we expect to uncover further targets with translational potential. These preclinical data might stimulate interest in translational studies in human disease.

It would be misguided to over-interpret findings from animal studies in terms of the translatability of EE research to human conditions. With regard to immune regulatory effects of EE, the majority of current research focuses on characterizing immune phenotypes in animal models and elucidating the underlying mechanisms. Our discussions on the implications of EE for human health and disease are somewhat speculative, but aim to stimulate interest in research of this area. On the other hand, appreciation of the complex environmental impacts on immunity is important for conducting and interpreting preclinical studies. As such, EE can serve as a valuable paradigm.

## Author Contributions

RX, SA, MC, and LC wrote and revised the manuscript. All authors contributed to the article and approved the submitted version.

## Funding

This work was supported by NIH grants CA166590 and AG041250 to LC, and CA210087 and CA163205 to MC.

## Conflict of Interest

The authors declare that the research was conducted in the absence of any commercial or financial relationships that could be construed as a potential conflict of interest.

## Publisher’s Note

All claims expressed in this article are solely those of the authors and do not necessarily represent those of their affiliated organizations, or those of the publisher, the editors and the reviewers. Any product that may be evaluated in this article, or claim that may be made by its manufacturer, is not guaranteed or endorsed by the publisher.
